# Epidemiology of central nervous system germ cell tumors: global patterns, geographic variation, and methodological challenges

**DOI:** 10.3389/fonc.2026.1962617

**Published:** 2026-10-21

**Authors:** Hirokazu Takami, Matthew M. K. Shing

**Affiliations:** 1 Department of Neurosurgery, The University of Tokyo, Tokyo, Japan; 2 Department of Pediatrics and Adolescent Medicine, Hong Kong Children’s Hospital, Department of Paediatrics, The Chinese University of Hong Kong, Hong Kong, Hong Kong SAR, China

**Keywords:** central nervous system germ cell tumor, epidemiology, geographic variation, germinoma, incidence

## Abstract

Central nervous system germ cell tumors (CNS GCTs) are rare neoplasms that predominantly affect children and adolescents and exhibit striking geographic and demographic variation. Although they account for only a small proportion of primary brain tumors worldwide, their incidence is consistently higher in East Asian populations than in Western countries; CNS GCTs represent only 2-5% of pediatric brain tumors in Western populations, whereas they account for approximately 8-20% in East Asia. CNS GCTs also demonstrate a marked male predominance and a characteristic age distribution with peak incidence during adolescence, with a high male-to-female ratio. In this review, we summarize current knowledge on the epidemiology of CNS GCTs, focusing on global incidence patterns, regional and demographic differences, and temporal trends reported in population-based studies. Comparative studies across Asian and Western cohorts have suggested geographic variation not only in disease frequency but also in clinical presentation, including differences in tumor location distribution. Potential risk factors are also discussed; recent genomic studies have identified susceptibility loci at the *BAK1* enhancer region, whose higher allele frequency in East Asian populations may partially contribute to the observed geographic disparity. In addition, we highlight the methodological challenges associated with studying this rare tumor, including limitations of cancer registry data, differences in diagnostic criteria and reporting across countries, especially the difficulty of distinguishing germinoma from non-germinomatous GCTs in large datasets. By integrating findings from international registries and recent epidemiological studies, this review aims to provide a comprehensive overview of the current landscape of CNS GCT epidemiology and to identify key gaps that should be addressed through future multinational collaborative research.

## 1 Introduction

Central nervous system germ cell tumors (CNS GCTs) are rare neoplasms that arise mainly from midline structures, most commonly the pineal and suprasellar regions, in children, adolescents, and young adults. Histologically, CNS GCTs are broadly divided into germinoma, which is mostly sensitive to radiotherapy and chemotherapy with excellent long-term survival, and non-germinomatous germ cell tumors (NGGCT), a heterogeneous group comprising teratoma, embryonal carcinoma, yolk sac tumor, choriocarcinoma, and mixed histologies that generally carries a less favorable prognosis (1, 2). Despite representing only a small fraction of all primary CNS tumors, with reported overall incidence on the order of 0.08 to 0.21 cases per 100,000 population depending on the population studied (3, 4), CNS GCTs disproportionately affect children and adolescents, in whom they account for 3 to 15% of primary pediatric brain tumors, and they demonstrate one of the most striking examples of geographic and demographic variation among CNS neoplasms (5).

Since the earliest large case series (6, 7), it has been recognized that CNS GCTs cluster along the midline neuraxis, occur with a pronounced male predominance, and peak in incidence around puberty. Subsequent population-based studies have consistently reported a substantially higher incidence in East Asian countries including Japan, South Korea, mainland China, Taiwan, and Hong Kong than in Western Europe, North America, or Oceania (8, 9). Whether this disparity reflects genetic susceptibility, environmental exposure, diagnostic and reporting practices, or some combination of these factors has remained incompletely understood.

In this review, we synthesize the current epidemiological literature on CNS GCTs, with attention to (i) global and regional incidence patterns drawn from population-based cancer registries; (ii) demographic and clinical variation, including tumor location and its geographic distribution; (iii) emerging evidence for genetic susceptibility, particularly at the *BAK1* locus; and (iv) the methodological challenges inherent to studying a rare tumor whose classification and case ascertainment vary across registries and countries. We conclude by identifying gaps that we believe should be prioritized in future multinational collaborative research.

## 2 Global incidence patterns

### 2.1 Incidence in Western populations

The most comprehensive population-based data on CNS GCT incidence in the West come from the Central Brain Tumor Registry of the United States (CBTRUS) and the Surveillance, Epidemiology, and End Results (SEER) program. In the most recent CBTRUS pediatric report, covering children and adolescents aged 0–19 years diagnosed between 2017 and 2021, CNS GCTs accounted for 814 cases (an average of 163 per year), corresponding to an average annual age-adjusted incidence rate (AAAIR) of 0.20 per 100,000 (95% CI 0.19–0.21), with a pronounced male predominance (male AAAIR 0.27 vs. female 0.12) (3). Across all ages, the CBTRUS 2018–2022 report similarly documents CNS GCT as a rare entity relative to gliomas and meningiomas, which dominate the all-age burden of CNS tumors (4).

A dedicated CBTRUS-based analysis covering 2006–2015 reported age-adjusted incidence rates of 0.111 per 100,000 in males and 0.038 per 100,000 in females, again demonstrating a strong male excess (8). A focused analysis of non-pineal CNS GCT using 2000–2004 CBTRUS and 1992–2005 SEER data found that a substantial proportion of non-pineal cases (31.6%) were registered only as “brain, not otherwise specified,” underscoring a site-classification issue discussed further in Section 5 (10).

Data from Europe are comparatively sparse. The Gironde CNS Tumor Registry in France, while not GCT-specific, documented an overall rise in CNS tumor incidence between 2000 and 2007 (annual percent change +2.33%), attributable in large part to improved detection through advances in neuroimaging rather than a true increase in tumor occurrence (11). In Australia, a national registry-based study of GCTs across all sites and ages (1982–2011) identified 552 pediatric cases and found that intracranial and other extragonadal sites accounted for roughly half of tumors in this age group, with incidence trends for some GCT subtypes stabilizing or declining over the study period (12).

### 2.2 Incidence in East Asia

In contrast, multiple national and hospital-based series from East Asia report substantially higher relative frequencies of CNS GCT among pediatric brain tumors, in the range of 8–20%, compared with 2–5% typically reported in Western series (13–15). The 13th report of the Brain Tumor Registry of Japan (2001–2004), which includes both pediatric and adult cases, recorded pure germinoma, HCG-producing germinoma, and other germ cell histologies together comprising approximately 2.3% of all primary CNS tumors (Committee of the Brain Tumor Registry of Japan, 2014).

A direct comparison of four Japanese and US tumor registries (the Japan Cancer Surveillance Research Group, SEER, the Brain Tumor Registry of Japan, and the US National Cancer Data Base) found that the age-standardized incidence of malignant CNS GCT in Japan (0.096 per 100,000; males 0.143, females 0.046) was not statistically significantly different from that in the United States (0.075 per 100,000; males 0.118, females 0.030) (16). This finding that national aggregate incidence in Japan and the US did not differ significantly stands in apparent tension with the broader East-Asia-versus-West narrative, and is best reconciled by considering ethnicity-specific rather than nationality-specific comparisons (Section 3.3).

In South Korea, a nationwide population-based study of childhood brain tumors (2005–2014) found that the overall age-standardized incidence of childhood brain tumors was slightly lower in South Korea than in the US (5.08 vs. 5.57 per 100,000); however, CNS GCTs were the most common childhood brain tumor subtype in the 10–14-year age group (age-standardized rate 0.78), and the authors concluded that a high incidence of CNS GCTs was a distinct feature of South Korean children and adolescents relative to Western populations (17).

Unpublished data from our group (Hong Kong Paediatric Haematology and Oncology Study Group [HKPHOSG] and Hong Kong Cancer Registry, 2020–2024) show an age-standardized incidence of CNS GCT among children aged 0–19 years of 0.50 per 100,000 (males 0.76, females 0.22; male-to-female ratio 3.45). These rates are higher than those reported for the United States by CBTRUS (3), particularly in males; in the 10–14- and 15–19-year age groups, rates in Hong Kong were 3.2–3.3 times those in the United States.

Population-based confirmation of the East Asian excess is also available from Taiwan. An analysis of the nationwide Taiwan Cancer Registry from 1995 to 2009, including individuals aged 0–19 years, reported an age-standardized incidence of CNS GCTs of 2.17 per million person-years. Incidence was higher in males than in females (2.87 vs 1.41 per million person-years; male-to-female incidence rate ratio, 2.03) and was approximately 1.3-fold higher than the corresponding US SEER estimate (1.6 per million person-years). The study also reported a significant annual increase in the incidence of germ cell neoplasms overall (annual percentage change, 2.3%; 95% CI, 0.8–3.9), although site-specific temporal trends for CNS GCTs were not provided (18).

Singapore, a Southeast Asian country with a predominantly Chinese population, provides an informative comparison. In a 15-year dual-institutional series from Singapore’s two tertiary pediatric neurosurgical centers (2002–2017), GCTs accounted for 44 of 396 pediatric brain tumors (11.1%) (19). This hospital-based proportion was reported to be similar to those in selected East Asian series and higher than in selected Western series, although it is not directly comparable with population-based incidence estimates. Singapore was not included in IICC-3, but Chinese and Malay datasets were included in IICC Volumes I and II.

Population-based CNS GCT-specific estimates are available from six Chinese registries in IICC-3 (1990–2013), with age-standardized incidence rates of 2.1 per million at ages 0–14 years and 2.2 per million at ages 0–19 years. Estimates varied across individual registries, although differences in registry coverage, observation period, and small case numbers preclude inference regarding true geographic variation. Available national registry data for mainland China from 2005 to 2017 showed a significant increase in the incidence of childhood CNS tumors overall in rural areas (annual percentage change, 6.2%; 95% CI, 2.4–10.2), but not in urban areas (20). Histology-specific information was not available in these national registry reports; therefore, whether CNS GCT incidence varies systematically by province, geographic region, or urban/rural residence remains unknown.

Beyond these national registry comparisons, regional and institutional pediatric series report broadly consistent frequencies across East Asia, including Japan (15.1% (14)), Taiwan (14.0% (15)), South Korea (11.2% (21)), and Hong Kong (19.8%, unpublished). Together, these registry- and series-based data support a consistent, though not perfectly uniform, elevation of CNS GCT incidence across East Asian populations, with the most robust and internally consistent comparative data, spanning Japan, the United States, and Hong Kong, placing Hong Kong at the upper end of this range.

Notably, this elevated incidence does not extend uniformly across Asia: a large multi-institutional series from India which was the first of its kind from South Asia, found that CNS GCT accounted for only 0.43% of all CNS tumors across three tertiary centers, a frequency the authors explicitly described as “similar to that in the West” rather than East Asia, despite India’s geographic proximity to, and shared “Asian” categorization with, the East Asian countries discussed above (13).

To provide a common registry-based framework for international comparison, we additionally reviewed the electronic results of International Incidence of Childhood Cancer, Volume III (IICC-3), which report age-standardized incidence estimates for the CNS GCT-specific ICCC-3 subgroup Xa ("intracranial and intraspinal germ cell tumors") by registry. Although differences in registry coverage, observation periods, and small case numbers limit formal comparisons, these data provide additional context for the marked international variation described above.

## 3 Geographic, demographic, and clinical variation

### 3.1 Age and sex distribution

Across virtually all reported series, CNS GCTs show a unimodal age distribution with an abrupt increase in incidence around puberty; in the original 389-case natural history series, 68% of patients were diagnosed between 10 and 21 years of age (7). This pattern is preserved in contemporary registry data, with incidence rising from a low baseline in early childhood to a peak in the 10–19-year age range (3, 22).

Male predominance is similarly near-universal, though its magnitude varies by tumor location and age. Overall male-to-female incidence rate ratios of approximately 2–4:1 are reported across most series (8, 10), with an even more pronounced excess, up to 15-fold, for tumors confined to the pineal region specifically (8, 23). Notably, this ratio appears relatively stable across the geographic settings compared to date (Hong Kong 3.5:1 at ages 0–19 years; Japan 3.1:1 and United States 3.9:1 for malignant CNS GCT at all ages (16)), suggesting that whatever mechanism underlies the East Asian excess in absolute incidence, it does not substantially alter the sex distribution of the disease.

GCTs arising at the typical adolescent age are considered counterparts of type II germ cell tumors, one of the seven pathogenetic categories of germ cell tumors. Type II germ cell tumors are thought to arise from primordial germ cells and show a marked male predominance (24). The biological basis for the male predominance of CNS GCTs, however, remains incompletely resolved. One hypothesis, extrapolated from the pathogenesis of gonadal GCTs, is that a permissive microenvironment associated with Y-chromosome material may contribute to tumor development. In particular, the testis-specific protein Y-encoded (TSPY) gene within the gonadoblastoma Y (GBY) region has been proposed as a candidate factor (25). In addition, the characteristic occurrence of GCTs around puberty raises the possibility that the pubertal hormonal milieu may modulate the survival, proliferation, or clinical emergence of ectopic germ cells. This concept has been invoked in the association between Klinefelter syndrome, impaired spermatogenesis, elevated gonadotropin levels, and GCTs (26, 27). These hypotheses are conceptually consistent with the BAK1/KIT-pathway and chromosomal-syndrome associations discussed in Section 4.

### 3.2 Tumor location and clinical presentation

CNS GCTs display a strong predilection for midline structures: in the classic series by Jennings et al. (7) series, 95% of tumors arose along the axis from the suprasellar (37%) to the pineal region (48%), with an additional 6% bifocal (both sites) at presentation. Histologic subtype correlated with location; the majority of germinomas (57%) arose in the suprasellar region, whereas most NGGCT (68%) arose in the pineal gland (p < 0.0001), a distinction with direct clinical implications, since suprasellar germinomas are particularly prone to prolonged pre-diagnostic symptomatic intervals, commonly presenting with diabetes insipidus and visual field defects that may precede radiographic diagnosis by months to years (28, 29).

Direct comparison of tumor location distribution between geographically distinct cohorts provides some of the clearest evidence that the East Asia–West disparity extends beyond raw incidence to clinical phenotype. In a comparison of a Mayo Clinic cohort (n=98) with a Japanese multi-institutional consortium cohort (n>200), basal ganglia location was significantly more frequent among Japanese cases (8.4% vs. 0%, p=0.008), while bifocal (neurohypophyseal and pineal) involvement was significantly more common at Mayo Clinic (18.8% vs. 5.8%, p=0.002), despite no significant difference in age or sex distribution between the cohorts (30). Basal ganglia and thalamic germinoma which is an atypical, non-midline presentation, appears to be disproportionately enriched among patients of East Asian descent (31), a finding that, raises the possibility that regional variation in incidence and in anatomical distribution share a common underlying basis rather than being independent phenomena.

### 3.3 Race- and ethnicity-based variation within multiethnic cohorts

Some of the strongest evidence that the East Asia–West disparity reflects population-level susceptibility rather than purely national, environmental, or healthcare-system factors comes from studies examining race and ethnicity within single multiethnic countries, principally the United States. Using SEER data from 1992 to 2010, intracranial GCT incidence in Asian/Pacific Islanders was significantly higher than in Whites, particularly in the 10–29-year age group (relative risk 2.05, 95% CI 1.57–2.64 in males; 3.04, 95% CI 1.75–5.12 in females) (9). A later, more granular analysis of CBTRUS/SEER data (2006–2015) that disaggregated the “Asian” category found marked heterogeneity: age-adjusted germinoma incidence was highest in Polynesians (2.246 per 100,000) and the “Other Asian” category (1.478), intermediate in Southeast Asians (0.837) and East Asians (0.702), and comparatively low in South Asians (0.111 per 100,000) (8). The most recent CBTRUS pediatric report similarly found non-Hispanic Asian or Pacific Islander children to have a 112% higher incidence of CNS GCTs than non-Hispanic White children (3).

This heterogeneity within the “Asian” category is directly corroborated by the India series discussed above, in which CNS GCT incidence among South Asian patients was low and resembled the West rather than East Asia (13). Ethnicity-stratified IICC-3 registry data from New Zealand and Hawaii provide further evidence that the broad Asian/Pacific Islander category conceals important population-specific variation. In New Zealand, Māori children and adolescents had the highest CNS GCT incidence and showed the expected male predominance, whereas Pacific peoples had elevated incidence at ages 10–14 years but an unexpected female-predominant pattern in the pediatric dataset. In Hawaii, incidence was likewise elevated and was largely borne by Asian and Pacific Islander children; Native Hawaiian-specific estimates were based on too few cases for stable interpretation (https://iicc.iarc.fr). Considered together with the essentially null Japan–US national comparison (16), these findings argue against a simple “Asian versus Western” dichotomy. Rather, they suggest that susceptibility varies substantially among ancestral populations, with elevated risk observed specifically in East Asian and some Pacific populations but not uniformly across all Asian or Pacific groups. Although the small ethnicity-specific case numbers warrant cautious interpretation, these patterns motivate ancestry-resolved epidemiological and genetic studies, including evaluation of whether variation in *BAK1* risk-allele frequency and effect contributes to differences in CNS GCT susceptibility (32).

## 4 Risk factors and genetic susceptibility

### 4.1 The *BAK1* locus

The first genome-wide association study (GWAS) specifically targeting CNS GCT was conducted in a Japanese cohort of 133 patients and 762 controls, and identified a common 4-base-pair deletion polymorphism (rs3831846) in an enhancer region adjacent to *BAK1* as significantly associated with disease risk (odds ratio 2.46, 95% CI 1.83–3.31; minor allele frequency 0.43 in the study population) (32). Functional reporter assays demonstrated that the risk allele attenuates enhancer activity, consistent with reduced *BAK1* expression as the likely mechanism. *BAK1* encodes a pro-apoptotic protein that is negatively regulated via the KIT/KITLG signaling axis during germ cell development; the authors proposed that diminished *BAK1* expression may impair the normal apoptotic elimination of ectopically migrated primordial germ cells, thereby permitting their persistence and eventual malignant transformation at extragonadal, including intracranial, sites. Critically for the geographic disparity discussed throughout this review, the risk allele is considerably more common in East Asian populations (approximately 49%) than in European populations (approximately 20%) (32), providing the first mechanistically plausible genetic explanation for the East Asian excess in CNS GCT incidence. The same variant is in strong linkage disequilibrium with a previously identified testicular GCT susceptibility locus, suggesting shared genetic architecture between gonadal and CNS GCT. Whether variation in the frequency and effect of the *BAK1* enhancer risk allele across ancestral populations contributes to the observed differences in CNS GCT incidence remains to be determined. Future ancestry-resolved genetic and epidemiological studies are needed to test this hypothesis.

### 4.2 Additional susceptibility loci and multi-ancestry findings

A subsequent multi-ancestry GWAS of pediatric GCTs (testicular, ovarian, CNS, and extragonadal combined; 1,927 cases, 10,601 controls) confirmed the association at *BAK1* and identified three additional genome-wide significant loci for GCT overall (*SPRY4*, *DMRT1*, *DEPTOR*), as well as six loci specific to the CNS GCT subgroup, including variants near *KIT*, *TFAM*, and *LZTR1 (*33). Notably, effect estimates for testicular and CNS GCT risk alleles were strongly correlated (Spearman’s ρ=0.63), reinforcing the concept, first suggested by the *BAK1* linkage disequilibrium finding, that CNS and gonadal GCT share substantial genetic etiology despite their markedly different anatomical origin. Earlier whole-exome and targeted sequencing of 124 CNS GCTs had already identified frequent, mutually exclusive somatic mutations in the MAPK pathway (48.4% of cases, most commonly in *KIT* [27.4%]) and, less commonly, the PI3K pathway (12.9%, particularly *MTOR* [6.5%]) (34), consistent with the central role of KIT-pathway signaling suggested by the *BAK1* findings above.

### 4.3 Syndromic associations

Beyond common polymorphisms, several case series and one case-control study have reported an excess of CNS GCT among patients with chromosomal syndromes, most notably Down syndrome and Klinefelter syndrome. An early series from Hong Kong described two children with Down syndrome among eight children with intracranial GCT treated over a defined period, both presenting at atypical, non-midline sites including basal ganglia and cerebellum and proposed that the co-occurrence was unlikely to be coincidental (35). This association has since been placed on firmer epidemiological footing: in a case-control study nested within the Germ Cell Tumor Epidemiology Study, syndromic chromosomal birth defects (principally Down and Klinefelter syndrome) were associated with a markedly increased risk of pediatric GCT overall (odds ratio 10.4, 95% CI 4.9–22.1), an association confined to males and not observed for nonsyndromic birth defects (36). Klinefelter syndrome has also been repeatedly linked to intracranial germinoma in case reports, including at atypical locations such as the medulla oblongata (37). While these syndromic associations account for only a small minority of overall CNS GCT cases, they support a broader model in which germ cell tumorigenesis is sensitive to genetic dosage effects, whether at the level of common regulatory polymorphisms such as *BAK1* or chromosomal copy-number abnormalities, converging on disrupted primordial germ cell survival or migration as a shared pathogenic mechanism.

## 5 Methodological challenges in CNS GCT epidemiology

### 5.1 Rarity and registry limitations

With incidence rates on the order of 0.1–0.2 per 100,000, CNS GCT is sufficiently rare that individual national registries, let alone single-institution series, are typically underpowered to detect anything but the largest effect sizes, and confidence intervals around subgroup-specific rates (by age, sex, race, or histology) remain wide even in the largest available datasets, including CBTRUS (3, 4). This has practical consequences beyond statistical power: as the South Korean nationwide study explicitly noted, the widely used International Classification of Childhood Cancer (ICCC) does not classify CNS GCTs as distinct CNS tumor categories, which the authors identified as a major obstacle to cross-study and cross-national comparison, alongside the more general absence of a single, universally applied case definition for childhood brain tumors (17).

### 5.2 Diagnostic and classification heterogeneity

Case ascertainment for CNS GCT is further complicated by inconsistent site coding: in one CBTRUS-based analysis, nearly one-third (31.6%) of nonpineal GCT cases were registered only under the nonspecific code “brain, not otherwise specified,” obscuring their true anatomical origin within aggregate registry statistics (10). Because CNS GCT can arise virtually anywhere along the midline neuraxis and, as discussed above, atypical locations such as the basal ganglia appear to be differentially represented across populations, incomplete or inconsistent site coding risks both undercounting the tumor overall and distorting comparisons of anatomical distribution between registries with differing coding practices.

### 5.3 Distinguishing germinoma from non-germinomatous GCT

Perhaps the most consequential methodological challenge specific to this tumor group is the reliable distinction between germinoma and NGGCT within large, registry-level datasets. The two entities carry markedly different prognoses and are managed with different treatment intensities, yet the proportion of cases diagnosed without histological confirmation varies enormously by data source. In a large US population-based registry series, 90.8% of registered germinomas and 86.9% of registered NGGCT were reported as histologically confirmed (8). By contrast, in the two major cooperative-group NGGCT trials, marker-based diagnosis without tissue confirmation was the norm rather than the exception: in the SIOP CNS GCT 96 study, only 76 of 149 patients (51%) underwent pretreatment surgical diagnosis, with the remainder diagnosed by protocol-defined marker thresholds (serum/CSF AFP >25 ng/mL and/or β-hCG >50 IU/L) alone (38); in the Children’s Oncology Group ACNS0122 trial, only 32 of 102 patients (31.4%) underwent resection or biopsy at diagnosis, with eligibility for the remainder based solely on marker elevation (39). This marker-based approach is internationally accepted and protocol-endorsed for NGGCT specifically, reflecting a deliberate effort to avoid unnecessary surgical morbidity in tumors with unambiguous marker elevation; it means, however, that a large proportion, in some series, the majority, of NGGCT cases in the clinical-trial and epidemiological literature have never been histologically verified. This introduces potential misclassification when data are aggregated across registries and trials with differing marker thresholds, differing assay availability, and differing practices for pursuing biopsy despite marker positivity (40–44). Because international comparisons of incidence, histological subtype distribution, and outcome all depend on consistent case definitions, harmonizing diagnostic criteria, or, at minimum, transparently reporting the proportion of histologically versus clinically diagnosed cases, represents an important target for future multinational registry collaboration.

## 6 Conclusion and future directions

The epidemiology of CNS GCT is characterized by a robust, if imperfectly uniform, elevation in incidence among East Asian populations relative to Western populations, a male predominance of similar magnitude across geographic settings, and a peak incidence around puberty that has been recognized since the earliest natural history studies. Contemporary population-based data, including previously unpublished data from Hong Kong presented in this review, extend this geographic picture beyond the historically emphasized Japan–US comparison and suggest that incidence may be even higher in some East Asian populations than earlier reports indicated. Importantly, disaggregated data from multiethnic cohorts and from South Asia demonstrate that this elevated risk is not a pan-Asian phenomenon but appears concentrated in East Asian, and possibly Pacific Islander, ancestry, a pattern that corresponds closely with the allele frequency of the *BAK1* enhancer risk variant, offering the first mechanistically plausible genetic explanation for a geographic disparity that had previously been attributed variably to genetics, environment, or ascertainment bias.

At the same time, this review highlights persistent methodological obstacles that complicate further progress: the rarity of the tumor limits the statistical power of any single national registry; inconsistent classification systems, exemplified by the ICCC's placement of CNS GCT within germ cell tumors (group X) rather than among CNS tumors (group III), hinder cross-national comparison; site-coding practices can obscure the true anatomical and geographic distribution of tumors; and the frequent reliance on marker- and imaging-based rather than histological diagnosis, while clinically reasonable, introduces heterogeneity into registry-level case definitions that is rarely made explicit.

Addressing these gaps will require continued and expanded international collaboration: harmonization of case definitions and site-coding practices across national registries; systematic incorporation of tumor marker and histological confirmation status into registry reporting; and, building on the genomic advances of the past several years, larger and more geographically and ethnically diverse genomic cohorts capable of resolving whether the *BAK1* locus and related variants fully account for the observed geographic disparity or whether additional genetic, epigenetic, or environmental contributors remain to be identified. Given the rarity of this tumor, such progress is unlikely to be achieved by any single country or consortium in isolation, underscoring the continued importance of the kind of multinational, multi-registry collaboration exemplified by the Japan–US and, now, Hong Kong comparisons summarized in this review.

## Figures and Tables

**Figure 1:**
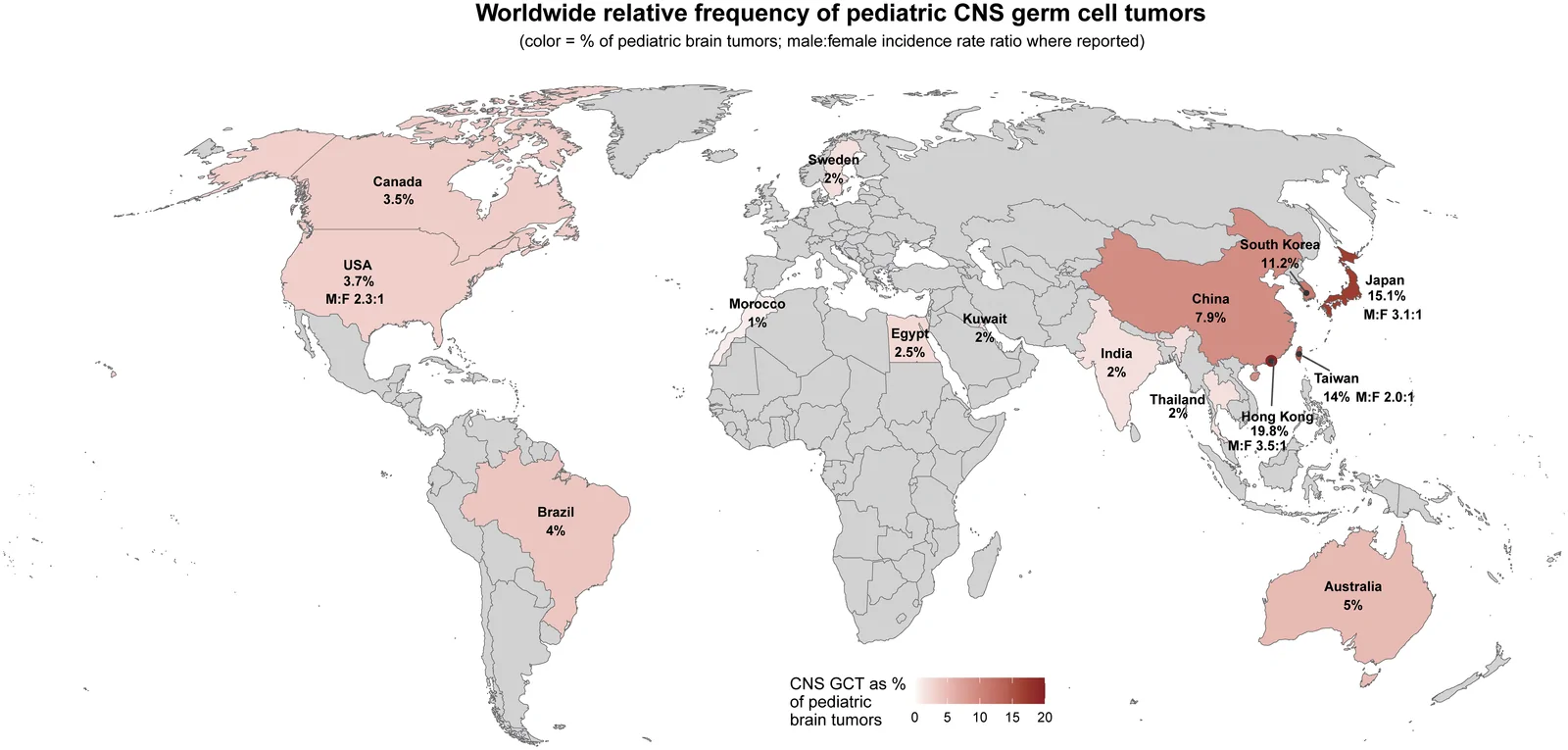
Worldwide relative frequency of pediatric CNS germ cell tumors. Adapted from Kakkar et al. (13), with three exact values added. Percentages are relative frequency (% of pediatric brain tumors), not absolute incidence. Hong Kong, Japan, and US values are exact; all other countries are as reported in Kakkar et al. (13). M:F ratios are derived from references (3, 16, 18) and unpublished Hong Kong data (see Sections 2.2 and 3.1).
